# Secreted NS1 proteins of tick-borne encephalitis virus and West Nile virus block dendritic cell activation and effector functions

**DOI:** 10.1128/spectrum.02192-23

**Published:** 2023-09-14

**Authors:** António A. R. Camarão, Olivia Luise Gern, Felix Stegmann, Felix Mulenge, Bibiana Costa, Babak Saremi, Klaus Jung, Bernd Lepenies, Ulrich Kalinke, Imke Steffen

**Affiliations:** 1 Institute of Biochemistry, University of Veterinary Medicine Hannover, Hannover, Germany; 2 Research Center for Emerging Infections and Zoonoses, University of Veterinary Medicine Hannover, Hannover, Germany; 3 Institute for Experimental Infection Research, TWINCORE, Centre for Experimental and Clinical Infection Research, a joint venture between the Helmholtz Centre for Infection Research and the Hannover Medical School, Hannover, Germany; 4 Institute for Immunology, University of Veterinary Medicine Hannover, Hannover, Germany; 5 Institute for Animal Breeding and Genetics, University of Veterinary Medicine Hannover, Hannover, Germany; 6 Cluster of Excellence—Resolving Infection Susceptibility (RESIST, EXC 2155), Hannover Medical School, Hannover, Germany; Regional Centre for Biotechnology, Faridabad, Haryana, India

**Keywords:** flavivirus, non-structural protein 1, tick-borne encephalitis virus, West Nile virus, dendritic cells, innate immunity, immune evasion

## Abstract

The flavivirus non-structural protein 1 (NS1) is secreted from infected cells into the circulation and the serum levels correlate with disease severity. The effect of secreted NS1 (sNS1) on non-infected mammalian immune cells is largely unknown. Here, we expressed recombinant sNS1 proteins of tick-borne encephalitis virus (TBEV) and West Nile virus (WNV) and investigated their effects on dendritic cell (DC) effector functions. Murine bone marrow-derived DCs (BMDCs) showed reduced surface expression of co-stimulatory molecules and decreased release of pro-inflammatory cytokines when treated with sNS1 of TBEV or WNV prior to poly(I:C) stimulation. Transcriptional profiles of BMDCs that were sNS1-exposed prior to poly(I:C) stimulation showed two gene clusters that were downregulated by TBEV or WNV sNS1 and that were associated with innate and adaptive immune responses. Functionally, both sNS1 proteins modulated the capacity for BMDCs to induce specific T-cell responses as indicated by reduced IFN-γ levels in both CD4^+^ and CD8^+^ T cells after BMDC co-cultivation. In human monocyte-derived DCs, poly(I:C)-induced upregulation of co-stimulatory molecules and cytokine responses were even more strongly impaired by TBEV sNS1 or WNV sNS1 pretreatment than in the murine system. Our findings indicate that exogenous flaviviral sNS1 proteins interfere with DC-mediated stimulation of T cells, which is crucial for the initiation of cell-mediated adaptive immune responses in human flavivirus infections. Collectively, our data determine soluble flaviviral NS1 as a virulence factor responsible for a dampened immune response to flavivirus infections.

**IMPORTANCE:**

The effective initiation of protective host immune responses controls the outcome of infection, and dysfunctional T-cell responses have previously been associated with symptomatic human flavivirus infections. We demonstrate that secreted flavivirus NS1 proteins modulate innate immune responses of uninfected bystander cells. In particular, sNS1 markedly reduced the capacity of dendritic cells to stimulate T-cell responses upon activation. Hence, by modulating cellular host responses that are required for effective antigen presentation and initiation of adaptive immunity, sNS1 proteins may contribute to severe outcomes of flavivirus disease.

## INTRODUCTION

The genus *Flavivirus*, within the family *Flaviviridae*, comprises 53 recognized species of enveloped, single-stranded positive-sense RNA viruses, many of which are important pathogens of public and animal health concern that collectively cause hundreds of millions of infections each year. Flaviviruses are primarily transmitted by arthropod vectors and include the most prevalent and widely distributed mosquito-borne viruses, the four dengue virus serotypes (DENV1-4) and West Nile virus (WNV), as well as one of the major tick-borne viral pathogens of humans, tick-borne encephalitis virus (TBEV) (1 – 4). Together, these viruses are responsible for some of the most pathogenic arbovirus infections, pose a serious threat to global health and have the potential to cause severe outbreaks. The clinical presentation of flavivirus infections may range from subclinical or self-limiting febrile illness to a spectrum of severe life-threatening conditions that include hepatitis, hypovolemic shock, encephalitis, acute flaccid paralysis, and congenital abnormalities (5, 6).

The flavivirus genome spans approximately 11 kb and encodes in a single open reading frame for a polyprotein that is post-translationally cleaved into three structural (C, prM/M, and E) and seven non-structural (NS1, NS2A, NS2B, NS3, NS4A, NS4B, and NS5) proteins. The first non-structural protein, NS1, is a conserved 46–55 kDa protein that exists in different glycoforms and distinct multifunctional oligomeric complexes. Dimeric NS1 is a membrane-associated glycoprotein essential for viral replication (7 – 9), whereas the hexameric form is secreted as a soluble high-density lipoprotein from infected cells (10 – 12). The secreted form of NS1 (sNS1) circulates in the host’s bloodstream during the acute phase of infection for several days (13 – 16) and has been implicated in numerous mechanisms of immune evasion and neuroinvasiveness (17). Complement system antagonism (18 – 20) and potential Toll-like receptor (TLR)-mediated signaling inhibition (21, 22) by sNS1 modulate cellular activation and inflammatory cytokine production, which may contribute to flavivirus pathogenesis. In this context, professional antigen-presenting cells (APCs), such as dendritic cells (DCs), are of particular relevance. DCs are critical regulators of the host response against viral infections that mediate initial pathogen sensing, antigen uptake, as well as antigen processing and presentation to T lymphocytes (23). In peripheral tissues, DCs sense RNA virus infections through pathogen recognition receptors (PRRs), such as the cytosolic retinoic acid-inducible gene-I (RIG-I)-like receptors (RLRs) and endosomal TLRs (24). Viral recognition leads to migration of the DCs to the regional draining lymph nodes, where they undergo a programmed phenotypic maturation in a process involving upregulation of MHC, adhesion proteins and co-stimulatory molecules, and cytokine production, which eventually results in enhanced presentation of viral antigens to naïve T cells. DCs appear to be common target cells that support flavivirus replication in the early stage of infection (25 – 29), thus providing the virus with opportunities to hijack DC effector functions as a host immune evasion strategy. Notably, many flaviviruses, including DENV, WNV, and Japanese encephalitis virus (JEV) are known to impair DC maturation and as a consequence to weaken efficient T-cell priming (30 – 37). However, the underlying mechanisms remain to be elucidated. Flaviviruses have developed different mechanisms to evade the early host innate immune response, which is a multifaceted process that primarily relies on the expression of non-structural proteins to inhibit antiviral interferon (IFN)-mediated responses. Previous studies have investigated the inhibition of innate immune pathways involved in WNV RNA sensing by intracellular overexpression of NS1 in mammalian cell lines (22, 38, 39). Further studies demonstrated that sNS1 proteins of WNV and DENV modulate the innate responses of primary DCs (21, 40).

Here, we specifically addressed immunomodulatory properties of sNS1 proteins of TBEV and WNV on primary DC effector functions. Although several studies showed that flaviviruses are capable of modulating DC maturation by inhibiting the surface expression of activation markers and/or production of cytokines, the specific viral determinants conferring this phenomenon have not been identified. We expressed and purified recombinant sNS1 proteins of TBEV and WNV and analyzed their function upon *in vitro* stimulation of primary DCs. We identified sNS1 as a viral factor that impairs innate responses of primary murine and human DCs. Functionally, the sNS1-mediated inhibition of DC activation resulted in reduced T-cell stimulation. Our findings indicate a new level of immune evasion mechanisms that are associated with flavivirus sNS1.

## RESULTS

### Recombinantly expressed flavivirus NS1 proteins are secreted as glycosylated high molecular weight oligomers

**Fig 1:**
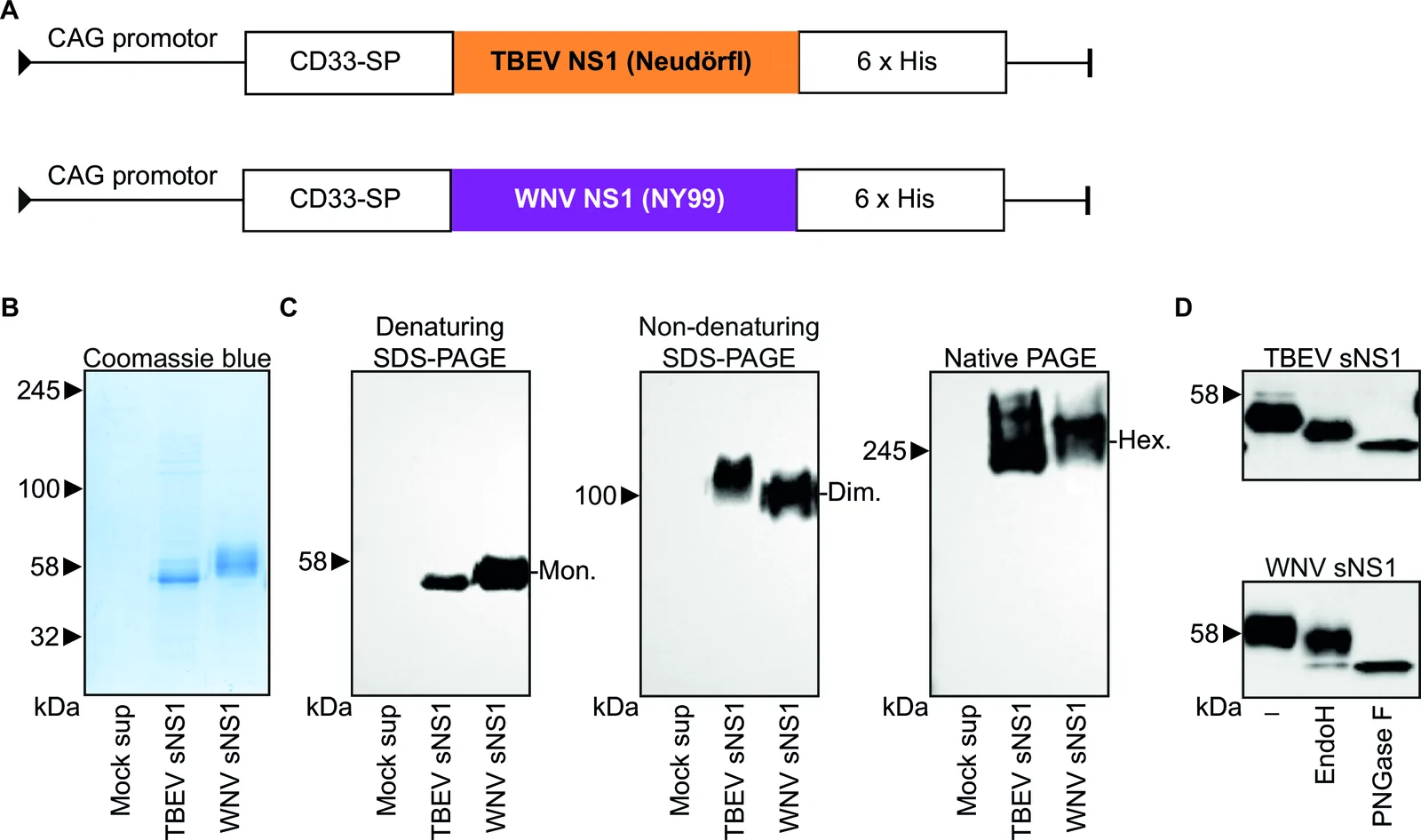
Cloning, expression, and purification of recombinant flavivirus secreted non-structural protein 1 (sNS1). (**A**) Schematic depiction of the expression constructs. (**B**) Coomassie blue staining of purified recombinant tick-borne encephalitis virus (TBEV) and West Nile virus (WNV) sNS1 proteins. One microgram total protein and the equivalent volume of mock supernatant (mock sup) was loaded on the gel. (**C**) Western blot analysis of purified recombinant sNS1 proteins. Left panel, fully denaturing conditions (heat and SDS) showing NS1 monomers (Mon.). Middle panel, non-denaturing conditions (SDS only) showing NS1 dimers (Dim.). Right panel, native conditions showing high molecular weight oligomers (Hex.). (**D**) Endoglycosidase digestion of purified recombinant sNS1 proteins. sNS1 was treated with EndoH or PNGase F or left untreated and analyzed by western blot. sNS1 proteins were detected using an anti-6x-His tag monoclonal antibody. Molecular weights are indicated in kDa.

Mammalian expression plasmids encoding the NS1 proteins of TBEV (Neudörfl) and WNV (NY99) were constructed. To enable efficient secretion and downstream purification, an N-terminal CD33 signal sequence and a C-terminal 6x-histidine tag (6x-His) were cloned in-frame with the respective NS1-encoding sequence (Fig. 1A). To ensure proper folding and post-translational modifications, the NS1 constructs were expressed in human embryonic kidney 293T cells (HEK293T). Cell culture supernatants containing recombinant sNS1 were harvested 48 hours post-transfection and subjected to immobilized metal affinity chromatography. Densitometric analysis of Coomassie blue-stained polyacrylamide gel electrophoresis (PAGE) showed a purity of at least 84% (Fig. 1B). Western blot analysis performed under fully denaturing conditions (SDS and heat) showed a band that corresponded to the 46–55 kDa monomeric form of NS1 (Fig. 1C, left panel), whereas under non-denaturing conditions (SDS only) the heat-labile, detergent-resistant dimeric forms at approximately 100 kDa were primarily observed (Fig. 1C, middle panel). Western blot analysis under native conditions indicated that only high molecular weight oligomers were present (Fig. 1C, right panel), further confirming the oligomeric state of the proteins. These data verified the oligomeric nature of the recombinantly expressed sNS1 proteins that resembles flaviviral NS1 secreted from infected cells (11, 41). The glycosylation profile of the purified recombinant sNS1 proteins was assessed by means of endoglycosidase digestion analysis. All proteins were sensitive to both digestion with endoglycosidase H (EndoH, removes high-mannose glycans) and peptide N-glycosidase F (PNGase F, removes high-mannose and complex glycans) as indicated by a gel shift in the western blot analysis (Fig. 1D). These data confirmed that the recombinant sNS1 proteins contained both high-mannose and complex glycans (42, 43).

### Flavivirus sNS1 modulates innate responses of primary murine bone marrow-derived dendritic cells

**Fig 2:**
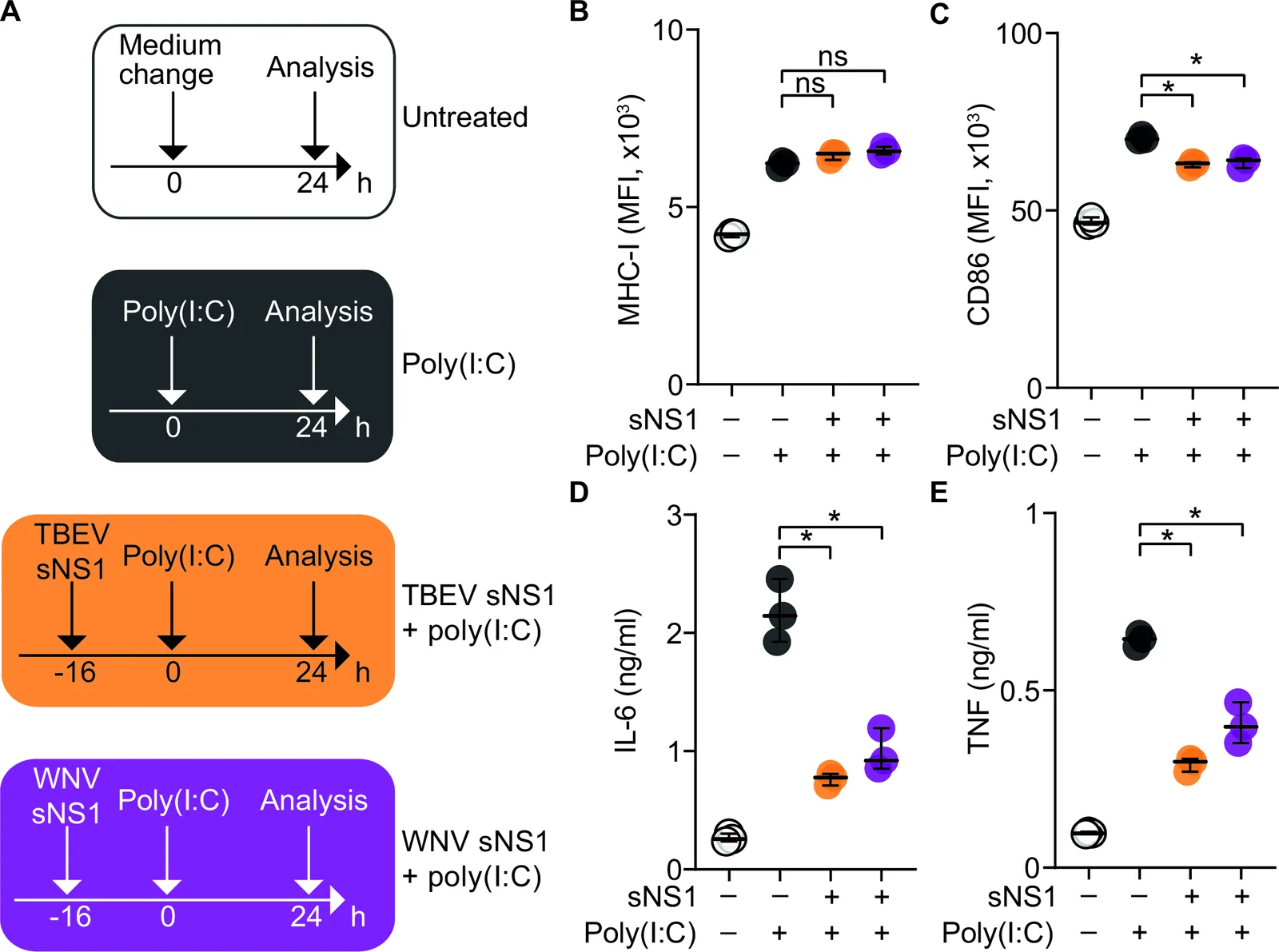
Flavivirus sNS1 inhibits innate responses in murine DCs. (**A**) Schematic depiction of the experiment. BDMCs were treated with 10 µg/mL recombinant tick-borne encephalitis virus (TBEV) secreted non-structural protein 1 (sNS1) or West Nile virus (WNV) sNS1 for 16 hours and stimulated with 25 µg/mL poly(I:C), were stimulated with poly(I:C) only or were left untreated. 24 hours post-stimulation, surface expression of MHC class I (**B**) and CD86 (**C**) was analyzed by flow cytometry, and secretion of IL-6 (**D**) and TNF (**E**) was measured by ELISA. Data shown are representative of at least three independent experiments. Statistical analyses were performed using a Student’s *t* test and asterisks indicate significant differences (ns, not significant; *P < 0.05).

sNS1 is found at particularly high levels (1 to 50 µg/mL) in the bloodstream of infected individuals (14, 15, 44), where it may be recognized and processed by circulating APCs. It was previously shown that WNV sNS1 alone did not induce cytokine responses or changes in surface expression of co-stimulatory molecules of primary murine bone marrow-derived macrophages (BMDMs) and DCs (BMDCs), respectively (21). However, upon myeloid cell stimulation by TLR agonists the secretion of IL-6 and IL-12 was significantly reduced in sNS1 treated BMDMs and BMDCs compared to control cells (21). We differentiated primary murine BMDCs from hematopoietic precursor cells obtained from the bone marrow of C57BL/6 mice (Fig. S1A). To investigate the effect of different sNS1 proteins on the innate responses of BMDCs, the cells were treated with 10 µg/mL of recombinant TBEV or WNV sNS1 protein for 16 hours. Subsequently, the cells were stimulated with polyinosinic–polycytidylic acid [poly(I:C)] for 24 hours (Fig. 2A). While the surface expression of MHC class I remained unaffected (Fig. 2B), the sNS1 treatment impaired poly(I:C)-induced expression of the co-stimulatory molecule CD86 (Fig. 2C). Furthermore, sNS1 treatment of BMDCs inhibited poly(I:C)-induced secretion of IL-6 (Fig. 2D) and TNF (Fig. 2E). Taken together, these data suggest that sNS1 inhibits innate responses of BMDCs, such as the expression of co-stimulatory molecules and cytokine production.

### Flavivirus sNS1 alters the transcriptional profile of BMDCs

**Fig 3:**
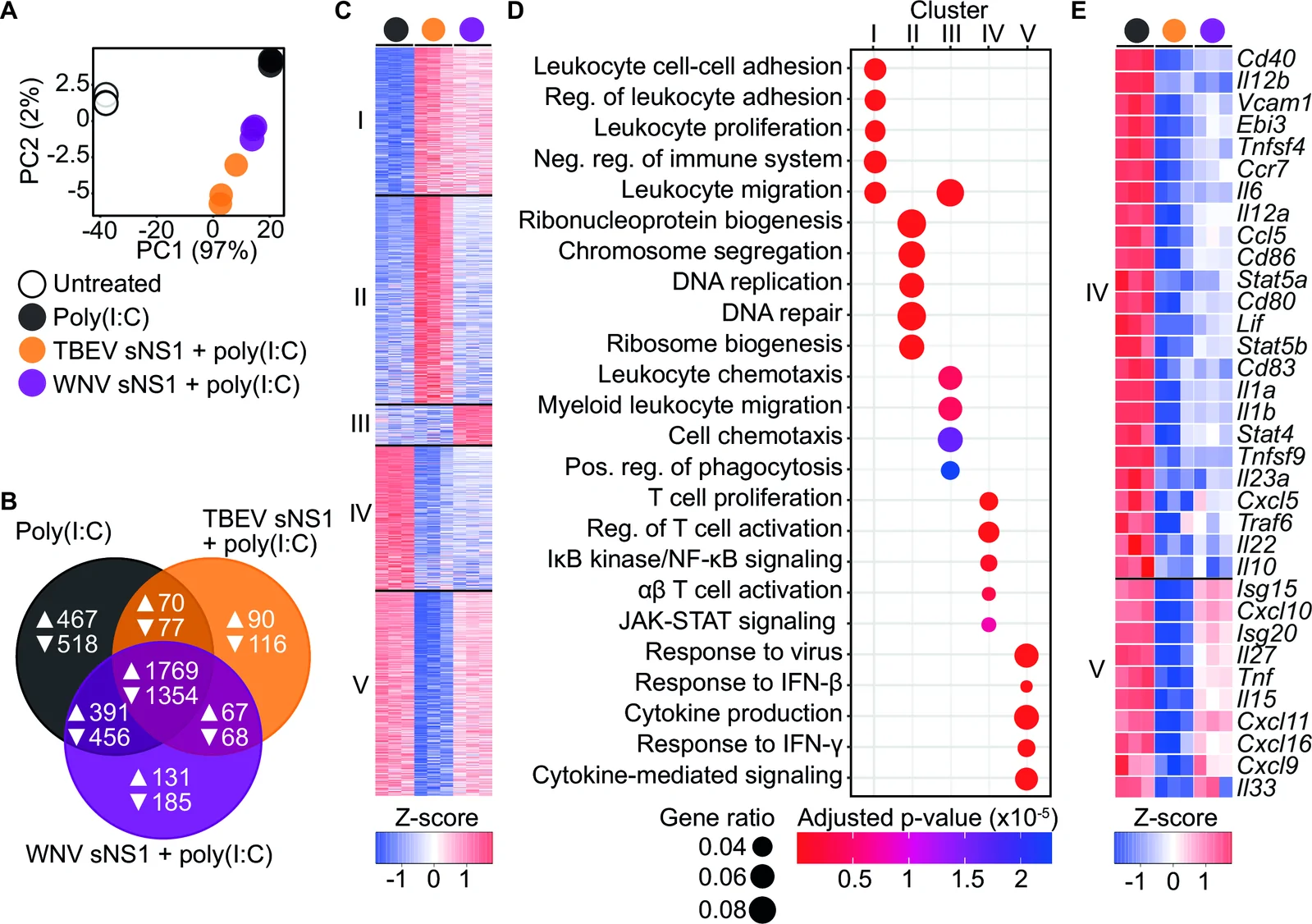
Flavivirus secreted non-structural protein 1 (sNS1) alters the transcriptional profile of murine DCs. BMDCs from C57BL/6 mice were treated with 10 µg/mL recombinant tick-borne encephalitis virus (TBEV) sNS1 or West Nile virus (WNV) sNS1 for 16 hours and stimulated with 25 µg/mL poly(I:C), were stimulated with poly(I:C) only or were left untreated (see Fig. 2A). At 24 hours post-stimulation, total RNA was isolated and RNA-seq analysis was performed. (**A**) PCA of untreated controls, poly(I:C)-stimulated, and TBEV sNS1 or WNV sNS1 pre-treated samples. (**B**) Venn diagram of differentially expressed genes (log_2_ fold-change > │1│, padj < 0.05) between either “poly(I:C)”, “TBEV sNS1 + poly(I:C)“ or “WNV sNS1 + poly(I:C)“ vs ”untreated“. (**C**) Heatmap of k-means clustering of DEGs (log_2_ fold-change > │1│, padj < 0.05). Each column represents transcripts from a technical replicate. (**D**) GO term enrichment analysis showing most enriched biological processes associated with each cluster of DEGs. The dot size indicates gene ratio defined as the number of genes within each GO term in comparison to the total number of upregulated genes. (**E**) Heatmap of k-means re-clustering of genes comprised in clusters IV and V.

Given the inhibitory effect observed on the innate immune response of sNS1-treated BMDCs, we performed transcriptional analyses by means of RNA sequencing (RNA-seq). Principal component analysis (PCA) of the transcriptome obtained from untreated controls, poly(I:C)-stimulated as well as TBEV sNS1 or WNV sNS1 pre-treated and poly(I:C)-stimulated BMDCs revealed distinct clusters (Fig. 3A). As expected, untreated controls clustered separately from the “poly(I:C)”, “TBEV sNS1 + poly(I:C)”, and “WNV sNS1 + poly(I:C)” groups. Similarly, although to a lesser extent, “TBEV sNS1 + poly(I:C)” and “WNV sNS1 + poly(I:C)” samples formed separate clusters. Using the selection criterion of log_2_ fold-change > │1│ and padj < 0.05, differential expression analysis with Wald test between either “poly(I:C)”, “TBEV sNS1 + poly(I:C)”, or “WNV sNS1 + poly(I:C)” versus the “untreated” group showed the highest numbers of differentially expressed genes (DEGs) in BMDCs treated with poly(I:C) alone, whereas TBEV sNS1 or WNV sNS1 pre-treatment prior to poly(I:C) stimulation resulted in a decrease of DEGs (Fig. 3B). Using unbiased k-means clustering of DEGs between “TBEV sNS1 + poly(I:C)”, “WNV sNS1 + poly(I:C)”, and “poly(I:C)” using likelihood ratio test revealed five different clusters (Fig. 3C). Cluster I comprised genes that were upregulated in “TBEV sNS1 + poly(I:C)” and “WNV sNS1 + poly(I:C)” samples. Cluster II and III consisted of genes that were exclusively upregulated in “TBEV sNS1 + poly(I:C)” or “WNV sNS1 + poly(I:C)”, respectively. Interestingly, cluster IV comprised genes that were downregulated in both sNS1 pre-treatments with subsequent poly(I:C) stimulation compared with samples stimulated with poly(I:C) only. Cluster V consisted of genes that were predominantly downregulated in “TBEV sNS1 + poly(I:C)” samples compared with the other experimental conditions. Using Gene Ontology (GO) terms to assess the biological function associated with cluster-specific DEGs, we revealed that cluster I comprised genes associated with leukocyte adhesion, proliferation, and migration. Cluster II contained genes involved in DNA replication and repair, whereas cluster III consisted of genes implicated in cell chemotaxis and myeloid leukocyte migration. Notably, clusters IV and V were comprised of genes associated with T cell proliferation, regulation of T cell activation, response to IFN-β, and cytokine-mediated signaling (Fig. 3D). In order to define genes mediating inhibition of innate immune responses through TBEV sNS1 or WNV sNS1 exposure in clusters IV and V, we retrieved genes based on their GO term annotations and performed k-means re-clustering. Of note, we identified cluster IV that comprised genes encoding for co-stimulatory molecules (*Cd40*, *Cd80*, *Cd83,* and *Cd86*), key pro-inflammatory cytokines (*Il1a*, *Il1b*, *Il6*, *Il12a,* and *Il12b*) and chemokines (*Ccl5* and *Cxcl5*) (Fig. 3E). In addition, cluster V consisted of genes encoding for cytokines (*Tnf*, *Il15, Il27,* and *Il33*), chemokines (*Cxcl9*, *Cxcl10*, *Cxcl11,* and *Cxcl16*), and IFN-stimulated genes (ISGs, *Isg15,* and *Isg20*) (Fig. 3E). Thus, the transcriptional profiles of BMDCs indicate that flavivirus sNS1 modulates the host gene expression associated with antiviral immune responses (Fig. S2).

### Flavivirus sNS1 modulates T-cell stimulation by BMDCs

**Fig 4:**
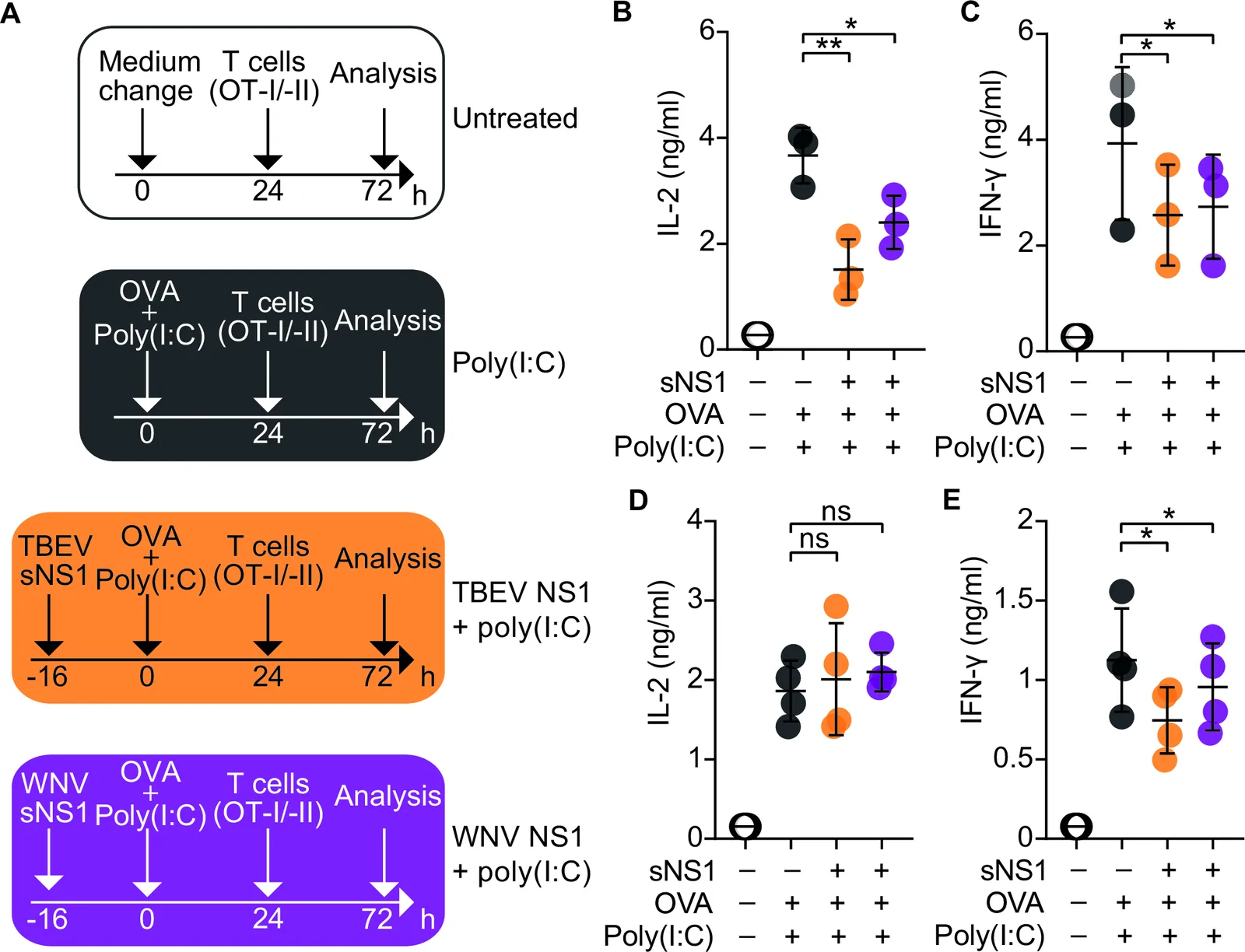
Treatment with flavivirus secreted non-structural protein 1 (sNS1) modulates activation of T cells by murine DCs. (**A**) Schematic depiction of the experiment. BMDCs from C57BL/6 mice were treated with 10 µg/mL recombinant tick-borne encephalitis virus (TBEV) sNS1 or West Nile virus (WNV) sNS1 and incubated with EndoGrade ovalbumin (OVA) and poly(I:C), were only treated with EndoGrade OVA and poly(I:C), or were left untreated. Negatively selected pan-T cells were isolated from OT-I or OT-II TCR-transgenic mice and co-cultured with stimulated BMDCs. After 48 hours, T cell-derived IL-2 and IFN-γ in co-cultures of BMDCs and OT-I TCR-transgenic CD8^+^ T cells (**B and C**) or OT-II TCR-transgenic CD4^+^ T cells (**D and E**) were determined by ELISA. Data shown are mean values of at least three biological replicates. Statistical analyses were performed using a Student’s *t* test and asterisks indicate significant differences (ns, not significant; *P < 0.05; **P < 0.005).

To investigate whether the sNS1-mediated modulation of DC functions affected their T-cell stimulatory capacity, we performed antigen presentation assays using BMDCs and T cells isolated from OT-I or OT-II TCR transgenic mice. In brief, BMDCs were incubated with TBEV sNS1 or WNV sNS1 and pulsed with the model antigen ovalbumin (OVA) simultaneous to poly(I:C) stimulation for 24 hours (Fig. 4A). Pan-T cells from OT-I or OT-II TCR-transgenic mice were isolated by negative selection (Fig. S1B). Then, T cells and activated BMDCs were co-cultured for 48 hours and T cell-derived IL-2 and IFN-γ levels determined by enzyme-linked immunosorbent assay (ELISA). Interestingly, sNS1 treatment of BMDCs significantly reduced IL-2 and IFN-γ responses of stimulated OT-I CD8^+^ T cells (Fig. 4B and C). In contrast, OT-II CD4^+^ T-cell stimulation did not show significant changes in IL-2 levels after BMDC incubation with the different sNS1 proteins (Fig. 4D), whereas the IFN-γ production was markedly decreased after pulsing with TBEV sNS1 or WNV sNS1 (Fig. 4E). Overall, these results suggest that the sNS1-mediated modulation of DC mediated T-cell stimulation has a major impact on the resulting T-cell effector functions.

### Flavivirus sNS1 inhibits innate responses of primary human monocyte-derived DCs

**Fig 5:**
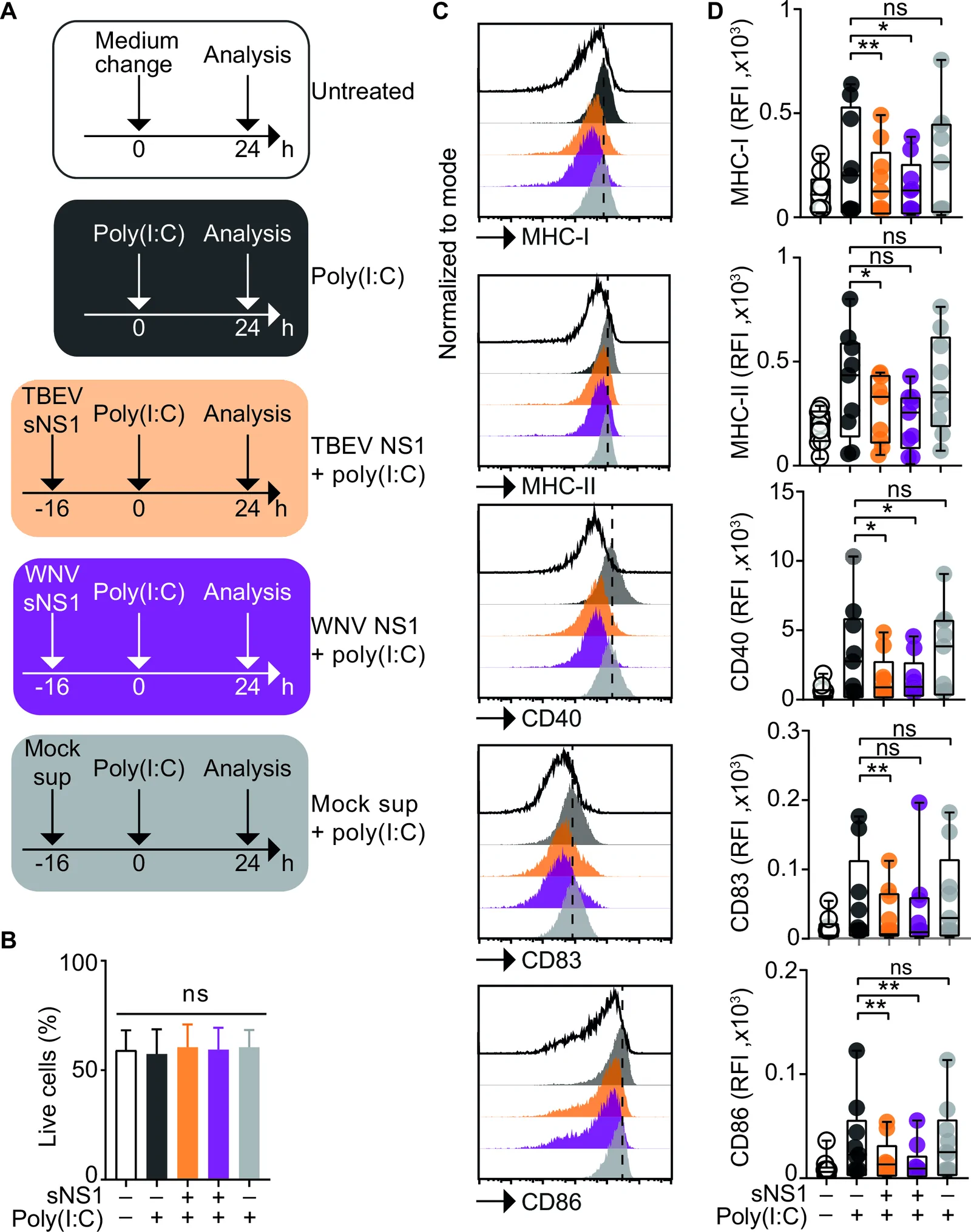
Flavivirus secreted non-structural protein 1 (sNS1) inhibits the maturation of moDCs. (**A**) Schematic depiction of the experiment. moDCs were pre-treated for 16 hours with 10 µg/mL recombinant tick-borne encephalitis virus (TBEV) sNS1 or West Nile virus (WNV) sNS1 or the same volume of mock supernatant (mock sup) and stimulated with poly(I:C), were only stimulated with poly(I:C) or were left untreated. Surface expression of DC markers MHC class I, MHC class II, CD40, CD83, and CD86 was determined by flow cytometry. (**B**) After 24 hours of stimulation with poly(I:C), cells were stained with Zombie-Aqua to determine the percentage of live moDCs following flavivirus sNS1 treatments. (**C**) Representative data from one human donor is shown in histograms for each treatment group and surface marker expression. (**D**) Quantified data from nine individual donors are displayed as relative fluorescence intensity (RFI), defined as the mean fluorescence intensity obtained from the specific antibody divided by that from the isotype control antibody (each data point represents a human donor). Statistical analyses were performed using a Wilcoxon signed rank test and asterisks indicate significant differences (ns, not significant; *P < 0.05; **P < 0.005).

**Fig 6:**
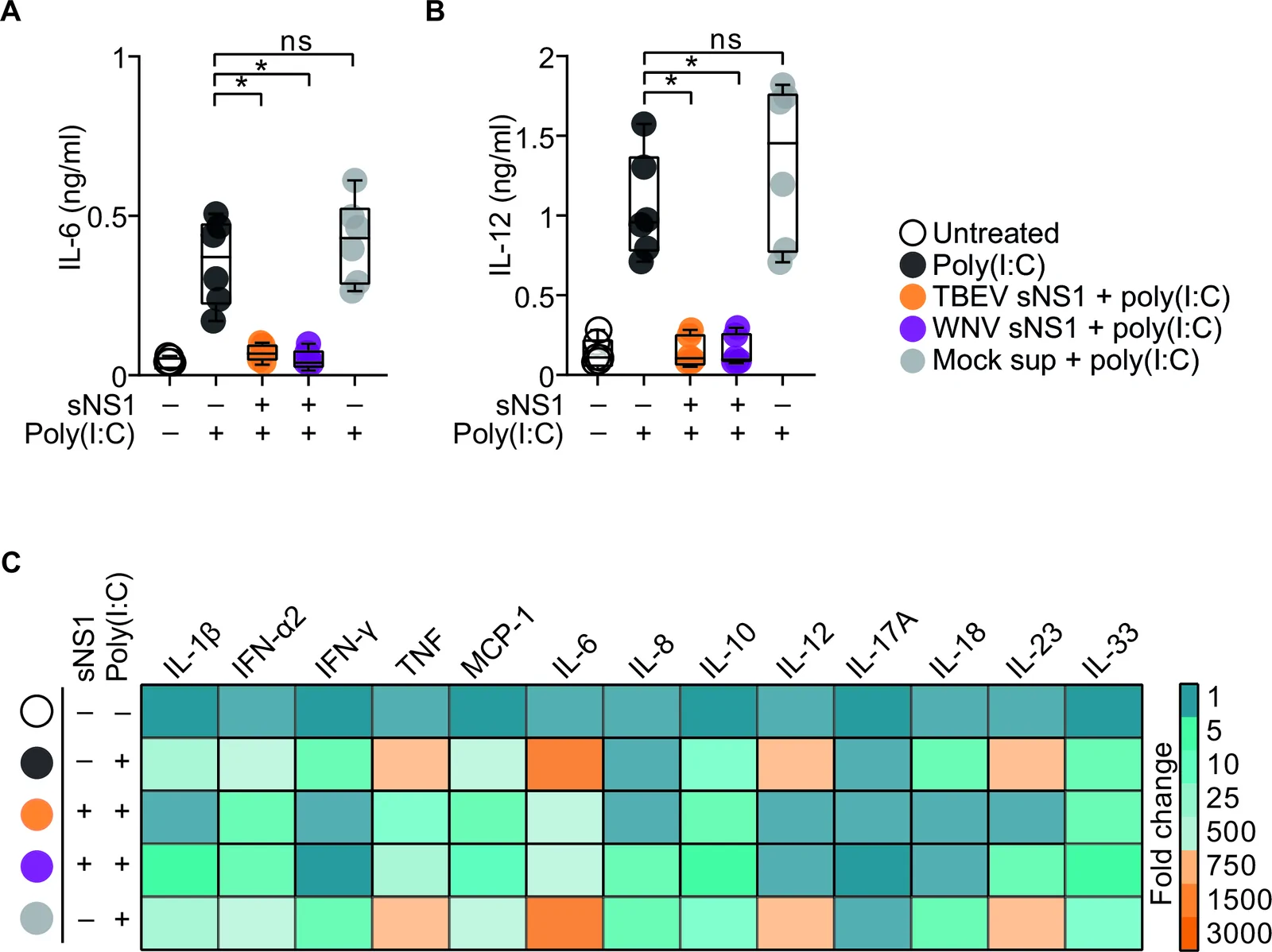
Flavivirus secreted non-structural protein 1 (sNS1) inhibits cytokine responses of moDCs. moDCs were treated as described in Fig. 5. At 24 hours post-stimulation, IL-6 (**A**) and IL-12 (**B**) secretion into cell culture supernatant was measured by ELISA (each data point represents a human donor). (**C**) Levels of inflammatory cytokines in moDCs supernatants were additionally detected by bead array. Cytokine levels are expressed as fold change over normalized mock (average of six different human donors). Statistical analyses were performed using a Wilcoxon signed-rank test and asterisks indicate significant differences (ns, not significant; *P < 0.05).

To validate the murine transcriptomic data at the protein level in a biologically relevant system, we used primary human monocyte-derived DCs (moDCs) that were obtained from peripheral blood mononuclear cells (PBMCs) isolated from blood of healthy donors (Fig. S3A and B). As before, moDCs were treated with 10 µg/mL of recombinant TBEV sNS1 or WNV sNS1 for 16 hours and then stimulated with poly(I:C) (Fig. 5A). We confirmed that the cell viability was not affected by the sNS1 pre-treatment (Fig. 5B) to ensure that any observed reduction of surface marker and cytokine expression was conferred by sNS1-mediated inhibitory effects. In fact, treatment with both sNS1 proteins substantially reduced the poly(I:C)-induced upregulation of MHC class I and II surface expression (Fig. 5C) and induction of the co-stimulatory molecules CD40, CD83, and CD86 (Fig. 5D). Furthermore, secretion of antiviral inflammatory cytokines and chemokines such as IL-1β, IFN-α2, IFN-y, TNF, MCP-1 (CCL2), IL-6, IL-10, IL-12, IL-18, and IL-23 was also inhibited in moDCs by treatment with TBEV sNS1 or WNV sNS1 prior to poly(I:C) stimulation when compared with moDCs stimulated with poly(I:C) only (Fig. 6A through C). Taken together, these data indicate that flavivirus sNS1 also inhibits key innate responses in human DCs.

## DISCUSSION

Herein, we demonstrate that recombinantly expressed sNS1 of TBEV and WNV modulate poly(I:C)-induced DC responses. We found that the transcriptional profiles of murine DCs treated with sNS1 prior to poly(I:C) stimulation showed downregulation of genes primarily involved in innate and adaptive antiviral immune responses. Consistent with this observation, sNS1 treatment of BMDCs affected their capacity to activate CD4^+^ and CD8^+^ T cells in an *in vitro* co-culture system. Finally, we validated the murine data at the protein level in primary human DCs. We observed a strong sNS1-mediated impairment of human DC effector functions, namely surface expression of activation markers and inflammatory cytokine production. Taken together, these results suggest an additional conserved role for flavivirus sNS1 in immune evasion through modulation of early host immune responses required for effective antigen presentation and initiation of adaptive immunity.

Flavivirus infections follow a biphasic course with an acute viremic phase, followed by viral clearance or invasion of immune-privileged sites, such as the central nervous system (CNS). High titer viremia during the acute phase of infection precedes neuroinvasion (45), suggesting an immune failure in the periphery in severe cases. Recognition and control of TBEV and WNV were shown to depend on the initiation of TLR (46, 47) and RLR (48 – 50) signaling pathways. In this study, we investigated poly(I:C) induced DC responses, which are dependent on TLR3 and RLR pathways, without inducing other relevant mechanisms involved in innate sensing of flaviviruses, such as cyclic GMP-AMP synthase (cGAS) (51, 52) and the NLR family pyrin domain containing 3 (NLRP3) inflammasome (53 – 56). Furthermore, poly(I:C) stimulation, in contrast to TBEV infection, does not cause cell damage. During the acute phase of flavivirus infection, sNS1 is secreted from infected cells (14, 57, 58) and high sNS1 levels in the serum of DENV-infected patients reportedly correlated with the development of severe disease (13, 15, 44), while high sNS1 antigenemia during WNV infection was associated with neuroinvasiveness (17). Coinciding with detectable viremia, circulation of sNS1 in the blood is transient and decreases with the onset of IgM production (16), suggesting an efficient capture of sNS1 by specific antibodies at later stages of infection. In fact, NS1 is highly immunogenic, induces protective antibody responses, and is being investigated as a subunit vaccine candidate for several flaviviruses (59, 60). While intracellular WNV NS1 has previously been proposed to play a role in the inhibition of the nucleic acid sensors TLR3 and RLRs (22, 39), a conserved function of NS1 proteins from different flavivirus species counteracting these pathways has been debated (61). An immunomodulatory role for secreted WNV NS1 leading to reduced cytokine secretion has previously been described in the murine system (21). In our study, we found evidence for immunomodulatory roles of TBEV sNS1 and WNV sNS1 in murine and human DCs. While inflammatory cytokine responses were downregulated to a similar extent in BMDCs pre-treated with either TBEV sNS1 or WNV sNS1, transcriptome analyses revealed differences between the two viral proteins in the modulation of immune signaling factors, such as ISGs. While TBEV sNS1 pre-treatment led to a muted induction of ISGs (*Isg15*, *Isg20*) after poly(I:C) stimulation, this effect was less pronounced in WNV sNS1 pre-treated BMDCs. Interestingly, WNV infection of moDCs has previously been reported to induce low expression levels of inflammatory cytokines/chemokines [IL-6, IL-12, TNF, and MCP-1 (CCL2)] and co-stimulatory molecules (CD40, CD80, and CD86) compared to the stimulation with a RIG-I agonist alone, whereas type I IFN induction was similar between WNV infection and RIG-I agonist treatment (62). Similarly, infection of DCs with a low pathogenic tick-borne flavivirus (Langat virus, LGTV) was reported to inhibit DC activation through defective IL-12 production and weak up-regulation of CD40, CD86, and MHC class II surface expression (63). Considering that TBEV sNS1 or WNV sNS1 treatment of DCs prior to poly(I:C) stimulation inhibited the expression of both MHC/co-stimulatory molecules (MHC class II, CD40, and CD86) and inflammatory cytokines/chemokines [IL-6, IL-10, IL-12, IL-18, IL-23, TNF and MCP-1 (CCL2)], we may speculate that sNS1 is involved in the reported flavivirus-mediated inhibition of DC effector functions.

While this study provides transcriptional, translational, and functional insights into the flavivirus sNS1-mediated downregulation of innate responses in primary murine and human DCs, further studies are necessary to address the molecular mechanisms underlying the capacity for sNS1 to inhibit DC effector functions. Considering the observed downstream effects of decreased surface expression of DC activation markers and dampened cytokine response, sNS1 is likely endocytosed by DCs. Indeed, it has been shown that sNS1 of WNV and DENV are internalized by murine and human DCs, respectively (21, 40). Recently, scavenger receptor B1 was described to interact with and mediate internalization of DENV sNS1 in hepatocytes (64), which to date, is the only *bona fide* receptor known to be involved in flavivirus sNS1 uptake.

The capacity of mosquito-borne flaviviruses to affect DC maturation and consequently influence T-cell priming or activation may be dependent on the viral species. In fact, it was shown that a WNV carrying a mutated NS1 with the substitution P101K, which is present DENV NS1, led to reduced WNV replication and neuroinvasion (17). Therefore, the NS1 protein might be a determining factor for the species-specific immunomodulatory and neuroinvasive capacity of flaviviruses. Viral tissue tropism might be an additional factor modulating DC activation. Systemic flaviviruses, such as the attenuated yellow fever virus vaccine strain 17D (65) or DENV (66), are able to induce multiple inflammatory mediators and the upregulation of co-stimulatory molecules, unlike highly pathogenic strains of neurotropic flaviviruses, such as WNV (NY99) (37) or Zika virus (ZIKV, strains PR-2015, P6-1966, MR-1947, and Dak-1984) (29), which exhibit rather immunomodulatory effects. Our data suggest a similar mechanism for highly pathogenic, neurotropic tick-borne flaviviruses, such as TBEV (Neudörfl). Importantly, dysfunctional T cell responses have been described in severe human cases of WNV neuroinvasive disease (67, 68), highlighting the importance of an effective transmission of innate immune signals into adaptive cell-mediated effector functions.

Cytokines, including IL-1β, IL-4, IL-6, IL-12, IL-23 and IL-33, are involved in the polarization of T cells, some of which were found to be dysregulated by sNS1 exposure of DCs prior to poly(I:C) stimulation. T cells exposed to IL-12 differentiate toward a T_H_1 phenotype, which initiates the killing of intracellular pathogens including viruses. IL-12 was prominently downregulated in DCs that were sNS1 pre-treated prior to poly(I:C) stimulation, relative to DCs stimulated only with poly(I:C)—both on the transcriptional and the protein level. IL-4 and IL-33 drive T_H_2 polarization of T cells, which promotes immunity against extracellular threats. IL-4 is produced by T_H_2 T cells as well as granuloctyes and IL-33 can be expressed by murine DCs, while human DCs do not express IL-33 (69). Indeed, *Il33* expression was induced in murine BMDCs upon poly(I:C) stimulation, which was dampened in TBEV sNS1 pre-exposed BMDCs. IL-6 and IL-23 induce the polarization of T cells towards a T_H_17 phenotype. Both IL-6 and IL-23 were upregulated upon poly(I:C) stimulation, but diminished in TBEV or WNV sNS1 pre-treated and poly(I:C)-stimulated DCs. A microenvironment showing low levels of IFN-γ and IL-12 may lead to reduced T_H_1 differentiation, while low IL-4 and low IL-6 levels may impair T_H_2 and T_H_17 differentiation, respectively. Generally, low levels of pro-inflammatory cytokines in the presence of IL-2 may indirectly favor the polarization of CD4^+^ T cells into a T regulatory (Treg) phenotype, which was previously shown to be enriched as a result of JEV infection of human moDCs (30, 31, 33, 34). Additionally, low levels of both IL-2 and proinflammatory cytokines in CD8^+^ T cells may result in dampened cytotoxic effector functions (31, 70). Our study proposes the sNS1 protein as a flaviviral factor that potentially impairs T-cell polarization. Based on our assay format, we cannot formally exclude any direct impact of sNS1 on the T cells. However, the sNS1-mediated modulation of DC innate immunity described in this study suggests a reduced capacity to stimulate T-cell responses. While the ability to downregulate the expression of key genes involved in immune activation and antigen presentation seems to be conserved between the sNS1 of TBEV and WNV, a set of chemokines attracting activated T cells (*Cxcl9*, *Cxcl10*, *Cxcl11,* and *Cxcl16*) were more potently downregulated in BMDCs upon TBEV sNS1 than WNV sNS1 treatment prior to poly(I:C) stimulation. These data imply that TBEV sNS1 might be more potent in inhibiting the chemoattraction of T cells by DCs than WNV sNS1, while both sNS1 proteins appear similarly potent in inhibiting DC activation and subsequently T-cell activation.

During the acute phase of flavivirus disease, high serum levels of sNS1 may not only exert direct immunomodulatory effects on infected DCs but may also induce paracrine immunosuppression, which would impair the antigen presenting potential of non-infected DCs. By reducing T-cell priming at the time of acute infection, circulating sNS1 may render the CNS more vulnerable to infection by other pathogens or inflammatory stimuli. The impairment of T-cell activation by sNS1-treated DCs may reduce T-cell responses that are required for effective flavivirus control. Further studies are needed to elucidate the *in vivo* relevance of our findings, especially in the context of NS1-based immunization strategies and secondary infections.

In summary, we present evidence that sNS1 is a key flaviviral factor responsible for the modulation of DC functions through inhibition of surface expression of DC activation markers and reduction of inflammatory cytokine/chemokine production. This study provides the base for further investigations on host immune evasion strategies employed by flaviviruses at the interface between innate and adaptive immune responses. Altogether, our study advances our understanding of how sNS1 contributes to immune evasion and flavivirus pathogenesis.

## MATERIALS AND METHODS

### Cloning, expression, and purification of recombinant secreted flavivirus NS1 proteins

The nucleotide sequences encoding the NS1 proteins of TBEV (Neudörfl, NC_001672) and WNV (NY99, NC_009942) were cloned into a pCAGGS mammalian expression vector in frame with an N-terminal CD33 signal sequence and a C-terminal polyhistidine tag (6x-His) using NEBuilder HiFi DNA Assembly (New England Biolabs, NEB). Recombinant NS1 constructs were transformed into DH5-alpha competent *Escherichia coli* cells (NEB), and the sequences were verified by Sanger sequencing. HEK293T cells were transiently transfected with the recombinant NS1 constructs or empty pCAGGS using a calcium phosphate transfection protocol (71). Culture supernatant was harvested 48 hours post-transfection and cleared by filtration (0.22 µm). Purification of 6x-His-tagged secreted native NS1 (sNS1) proteins was performed by immobilized metal affinity chromatography on HisTrap HP 1 mL columns (Cytiva). In brief, sNS1-containing supernatants were mixed with 5× binding buffer (100 mM sodium phosphate, 2.5 M NaCl, 200 mM imidazole, pH 7.4), and loaded onto the columns pre-equilibrated in binding buffer (20 mM sodium phosphate, 0.5 M NaCl, 40 mM imidazole, pH 7.4). Columns were washed with binding buffer and sNS1 eluted with elution buffer (20 mM sodium phosphate, 0.5 M NaCl, 500 mM imidazole, pH 7.4). Purified sNS1 was dialyzed against PBS at 4°C using Pur-A-Lyzer Maxi Dialysis Kit (MWCO 12–14 kDa, Merck) following the manufacturer’s recommendations. In parallel, the negative control, corresponding to the culture medium of empty pCAGGS-transfected HEK293T cells (mock supernatant) was subjected to the same purification and dialysis protocols. The purity and concentration of the dialyzed samples were assessed by Coomassie blue staining and Pierce BCA Protein Assay Kit (Thermo Fisher Scientific), respectively.

### Protein gel electrophoresis and western blotting

Recombinant flavivirus sNS1 proteins were denatured at 100°C for 10 minutes in an equal volume of 2× loading buffer (1 M Tris pH 6.8, 1 mM EDTA, 2% SDS, 5% β-mercaptoethanol, 10% glycerol, and 1% bromophenol blue), resolved in SDS-containing 10% polyacrylamide gel (SDS-PAGE), and electrically transferred to nitrocellulose membranes (Semi-Dry-Blotter MAXI, Carl Roth). Membranes were blocked for 1 hour at room temperature (RT) in Tris-buffered saline (TBS) containing 0.05% Tween-20 (TBS-T) with 5% skim milk and then probed with an anti-6x-His tag monoclonal antibody (1:5,000, clone J099B12, BioLegend), rocking overnight at 4°C. After extensive washes with TBS-T, membranes were incubated with HRP-conjugated goat anti-mouse IgG (1:5,000, clone Poly4053, BioLegend) for 1 hour at RT. Proteins were detected using a chemiluminescent substrate (SuperSignal West Pico, Thermo Fisher Scientific) and visualized using a ChemiDoc XRS + system (Bio-Rad).

The oligomeric status was analyzed by native PAGE. Non heat-denatured proteins in a loading buffer without SDS or β-mercaptoethanol were resolved by electrophoresis without SDS in the gel and running buffers. The glycosylation profile was assessed by endoglycosidase digestion analysis. Purified recombinant sNS1 proteins were digested with endoglycosidase H (Endo H, NEB) or peptide-N-Glycosidase-F (PNGaseF, NEB) for 1 hour at 37°C. Digested samples were analyzed by western blot.

### Cell culture

HEK293T cells were maintained in high-glucose Dulbecco’s modified Eagle’s medium (DMEM, Sigma) supplemented with 10% heat-inactivated fetal bovine serum (FBS, Corning), 10,000 U/mL penicillin, 10 mg/mL streptomycin, and 2 mM L-glutamine.

Primary murine BMDCs were obtained from tibia and femur bone marrows of C57BL/6 mice and cultured in Iscove’s modified Dulbecco’s medium (IMDM, PAN-Biotech) supplemented with 10% FBS, 2 mM L-glutamine, 100 U/mL penicillin, 100 µg/mL streptomycin, and 10% granulocyte-macrophage colony stimulating factor (GM-CSF) containing supernatant derived from X63 cells (72). Medium was exchanged every 48 hours and BMDCs were used after 8 days to ascertain that ≥80% of the cell population expressed the DC differentiation marker CD11c.

Primary human moDCs were obtained from PBMCs isolated from blood of healthy donors via Ficoll density gradient centrifugation. CD14^+^ monocytes were isolated by magnetic-activated cell sorting (Miltenyi Biotech) and differentiated into moDCs for 5 days in serum-free CellGenix GMP dendritic cell medium (CellGenix) supplemented with 1,000 U/mL GM-CSF (Miltenyi Biotech) and 1,000 U/mL interleukin-4 (IL-4, Miltenyi Biotech). All cells were grown at 37°C and 5% CO_2_ in humidified atmosphere.

### Dendritic cell stimulation assays

Differentiated BMDCs or moDCs were seeded at a concentration of 10^6^ cells/mL in 96-well plates and treated with 10 µg/mL of purified recombinant TBEV sNS1 or WNV sNS1 or an equal volume of supernatant from HEK293T cells transfected with the empty pCAGGS vector (mock sup), respectively, or left untreated. After 16 hours incubation, DCs were stimulated with 25 µg/mL polyinosinic-polycytidylic acid (poly(I:C), Bio-Techne) for 24 hours.

### Dendritic cell—T cell co-culture assay

Differentiated BMDCs were seeded at a concentration of 2 × 10^5^ cells/mL in 96-well plates and treated with 10 µg/mL of purified recombinant of TBEV sNS1 or WNV sNS1 or left untreated. After 16 hours incubation, DCs were stimulated with 25 µg/mL poly(I:C) and 0.3 mg/mL EndoGrade ovalbumin (Lionex) for 24 hours. T cells were isolated from spleens of OT-1 C57BL/6-Tg(TcraTcrb)1100Mjb/Crl and OT-2 B6.Cg-Ptprca Pepcb Tg(TcraTcrb)425Cbn mice (provided by Reinhold Förster, Hannover Medical School, Germany) by magnetic activated cell sorting using the Pan T Cell Isolation Kit II mouse (Miltenyi Biotec). Purified T cells were adjusted to 1 × 10^6^ cells/mL and co-cultured with pre-stimulated BMDCs for 48 hours.

### Cytokine ELISAs

Levels of murine IL-6 and TNF in cell-free supernatants from BMDCs, levels of murine IL-2 and IFN-γ in cell-free supernatants from BMDC—T cell co-culture and levels of human IL-6 and IL-12 in cell-free supernatants from moDCs were determined by ELISA according to the manufacturer’s protocol (Peprotech). Plates were developed with 3,3′,5,5′-tetramethylbenzidine (TMB) or 2,2′-azino-bis(3-ethylbenzothiazoline-6-sulfonic acid) (ABTS) and absorbance measured at 450 nm with a 620 nm wavelength correction or at 405 nm with a 650 nm wavelength correction, respectively, in a Multiskan GO microplate reader (Thermo Fisher Scientific).

### Bead-based cytokine array

Cell-free supernatants from moDCs were used to determine the human inflammatory cytokine profiles using a LEGENDplex Multianalyte Flow Assay Kit (BioLegend, cat#740809) following the manufacturer’s protocol. Quantification of cytokine concentrations was performed using a ID7000 Spectral Cell Analyzer (Sony), and data were analyzed with LEGENDplex software, version 8.0 (BioLegend).

### Flow cytometry

After stimulation experiments, BMDCs were washed twice with PBS containing 1% FBS and blocked with an anti-mouse CD16/32 (1:100, clone 93, Thermo Fisher Scientific) antibody for 10 minutes at 4°C. Cells were then stained with APC-conjugated anti-mouse CD11c (1:250 dilution, clone N418, Thermo Fisher Scientific), FITC-conjugated anti-mouse MHC class I (1:100 dilution, clone AF6-88.5.5.3, Thermo Fisher Scientific), and PE-conjugated anti-mouse CD86 (1:200 dilution, clone B7-2, Thermo Fisher Scientific) antibodies for 20 minutes at 4°C in the dark. After washing, cells were fixed with 1% paraformaldehyde and analyzed using an Attune NxT flow cytometer (Thermo Fisher Scientific). In all flow cytometry assays, BMDCs were hierarchically gated to exclude debris followed by single cell gating.

Negatively selected pan-T cells were tested for purity as previously described (73) and stained with PE-conjugated antimouse CD4 (1:200 dilution, clone GK.15, BD Biosciences) and APC-conjugated antimouse CD8 (1:200 dilution, clone 53–6.7, BD Biosciences) following the protocol described above and analyzed using an Attune NxT flow cytometer (Thermo Fisher Scientific).

MoDCs were washed in PBS and stained with Zombie-Aqua live/dead fixable dye (BioLegend). Unspecific immunolabeling conferred by Fc-receptor binding was blocked by the addition of human immunoglobulin (Privigen). For the characterization of the surface phenotype of moDCs, cells were immunolabeled with PacificBlue-conjugated anti-human CD14 (1:50, clone HCD14, BioLegend) and PE-Cy7-conjugated anti-human CD11c (1:50, clone 3.9, BioLegend). To determine the maturation status, moDCs were immunolabeled with PE-Cy5-conjugated anti-human MHC class I (1:50, clone W6/32, BioLegend), AF700-conjugated anti-human MHC class II (1:25, clone L243, BioLegend), BV421-conjugated anti-human CD40 (1:50, clone 5C3, BioLegend), PE-Dazzle-594-conjugated anti-human CD83 (1:50, clone HB15e, BioLegend), and BV605-conjugated anti-human CD86 (1:50, clone IT2.2, BioLegend) antibodies for 20 minutes at 4°C in the dark. Data were acquired using a ID7000 Spectral Cell Analyzer (Sony), and analysis was performed on FlowJo version 10 software (Tree Star Inc.).

### Transcriptome analysis

Murine BMDCs were harvested in Trizol (Thermo Fisher Scientific), and total RNA was isolated from BMDCs using Directzol RNA Miniprep Plus Kit (Zymo Research) according to the manufacturer’s protocol. Stranded, polyA-enriched, TruSeq RNA libraries were prepared and sequenced on Illumina NovaSeq platform with 100 bp paired-end read configuration (Microsynth). Quality control of the sequenced raw FASTQ files was performed with FastQC software (version 0.11.9) and mapped to Ensembl mouse genome reference version GRCm39 using STAR (74), yielding raw read counts per gene and sample. Genes with a maximum read count <10 in all samples were removed before further analysis. Normalization and differential gene expression analysis were carried out using the R-package DESeq2 (75). Differential expression analysis was performed pairwise between all experimental groups. GO term enrichment analysis was done using the R-package ClusterProfiler (76). Grouping of samples was explored using principal component analysis, and clustered heatmaps were used to visualize expression profiles of selected genes.

### Statistical analyses

Statistically significant differences of cytokine and surface marker expression values between all pairs of experimental groups were determined using a Student’s *t* test or Wilcoxon signed-rank test on GraphPad Prism (version 9, GraphPad). *P*-values ≤ 0.05 were considered to be statistically significant.

## Data Availability

All RNA sequencing data is available under the Gene Expression Omnibus (GEO) accession number GSE232775.

## SUPPLEMENTAL MATERIAL

Fig. S1 spectrum.02192-23-s0001.eps Surface marker expression of murine BMDC and T cells.

Fig. S2 spectrum.02192-23-s0002.eps Normalized gene counts for selected cytokines involved in T-cell polarization.

Fig. S3 spectrum.02192-23-s0003.eps Surface marker expression of human moDCs.

Supplemental Material Legends spectrum.02192-23-s0004.docx Captions for supplemental figures.

ASM does not own the copyrights to Supplemental Material that may be linked to, or accessed through, an article. The authors have granted ASM a non-exclusive, world-wide license to publish the Supplemental Material files. Please contact the corresponding author directly for reuse.
