# Supplementary material for: Secreted NS1 proteins of tick-borne encephalitis virus and West Nile virus block dendritic cell activation and effector functions
Source: Microbiol Spectr. 2023 Sep 14;11(5):e02192-23. doi: 10.1128/spectrum.02192-23 (PMC10581055; doi:10.1128/spectrum.02192-23)
Supplement: Supplemental Material Legends — Captions for supplemental figures. [file spectrum.02192-23-s0004.docx]

**Supplemental material**

**Figure legends**

**S1 Fig. Surface marker expression of murine BMDC and T cells.** Primary murine bone-marrow derived dendritic cells (BMDCs) were hierarchically gated to exclude debris, followed by single cell gating and gating on CD11c^+^ cells to determine purity and differentiation status (A). Negatively selected pan-T cells were isolated from spleens of OT-I or OT-II TCR-transgenic mice and hierarchically gated on T cells, single cells and CD4^+^ and CD8^+^ T cells (B).

**S2 Fig. Normalized gene counts for selected cytokines involved in T cell polarization.** Normalized gene counts of *Il1b*, *Il4*, *IL6*, *IL12a*, *Il23a*, *Il33*, *Tgfb2* and *Tnf* in untreated controls, poly(I:C)-stimulated and TBEV sNS1 or WNV sNS1 pre-treated BMDC prior to poly(I:C) stimulation as described in Fig. 2 and Fig. 3.

**S3 Fig. Surface marker expression of human moDCs.** Primary human monocyte-derived dendritic cells (moDCs). Cells were hierarchically gated to exclude debris, followed by single cell gating and live cell gating (A). DC differentiation is shown by the presence of CD11c and absence of CD14 (B).
